# Comparing shift work tolerance across occupations, work arrangements, and gender

**DOI:** 10.1093/occmed/kqad090

**Published:** 2023-08-17

**Authors:** I Saksvik-Lehouillier, T A Sørengaard

**Affiliations:** Department of Psychology, Norwegian University of Science and Technology, Trondheim, Norway; BI Norwegian Business School, Trondheim, Norway

## Abstract

**Background:**

There are individual differences in shift work tolerance; however, we lack knowledge about how this is experienced across different occupations, sex and shift types.

**Aims:**

The aim was to describe and investigate shift work tolerance, and individual differences in shift work tolerance, in two occupations, between men and women and between day/evening workers and rotating shift workers.

**Methods:**

Cross-sectional questionnaire study. The sample was comprised of 315 retail workers and 410 police employees.

**Results:**

Shift work tolerance was higher among police employees compared to retail workers, among men compared to women, and among day workers compared to evening/rotating shift workers. The difference was larger between occupations than between sex and shift type. Evening workers had more symptoms of shift work intolerance than rotating shift workers. Neuroticism and autonomy were related to all symptoms of shift work tolerance among retail workers, but not police employees.

**Conclusions:**

It is important to consider the type of occupation and the work context when tailoring work arrangements to the individual.

## Teaser

In this study, we describe and investigate different aspects of shift work tolerance to be able to provide guidelines for tailoring shift work to individual needs. The type of occupation is more important for how well individuals tolerate shift work than if they are men or women or if they workday, evenings or rotating shift work. Neuroticism and autonomy are important to shift work tolerance for retail workers, but not for police employees.

**Key learning points**

**What is already known about this subject:**

- Shift work intolerance is concerned with experiencing sleep problems, fatigue, physical functioning (e.g. digestive troubles) and increased sensitivity, and aggressiveness as a result of working outside normal daytime hours (6 a.m. to 7 p.m.).
- There are individual differences in shift work tolerance in terms of sex, age, personality and genetics.

**What this study adds:**

- The type of occupation may be more important for shift work tolerance than sex and type of shift.
- High autonomy and low neuroticism are related to better shift work tolerance in retail workers, but not in police employees.

**What impact this may have on practice or policy:**

- Sex differences in shift work tolerance should be interpreted with caution with attention to possible underlying mechanisms, especially in terms of occupation.
- Larger attention should be given to the characteristics of the workers in a specific occupation, content of work, work tasks and activity performed at work in relation to different symptoms of shift work tolerance. Hence, larger attention should be given to the behavioural factor of the two-process model of sleep, not only the homeostatic and circadian factors.
- New instruments measuring variables related to shift work tolerance should be examined, especially instruments that can replace HADS in measuring increased sensitivity and aggression.

## Introduction

Shift work refers to a work schedule where workers do not always work the same hours within a 24-hour period [1], and may include night work where most of the working time falls between 10 p.m. and 6 a.m. [2]. This type of work increases the risk of occupational accidents and is a serious risk factor for the health of the worker [3–6]. Shift work tolerance refers to an individuals’ resilience towards experiencing negative effects of shift work, that is, sleep problems, fatigue, physical functioning, and increased sensitivity and aggressiveness [7,8]. The concept has been studied for 40 years, and despite several efforts [9], we still do not have a clear definition or standard way of measuring it [10]. Specifically, we lack knowledge about how shift work tolerance is experienced across different occupations and types of shifts [10]. It has also been debated whether high shift work tolerance implies a general work tolerance regardless of shift type [11].

There are individual differences in shift work tolerance in terms of sex, age, personality and genetics [12,13]. However, the research on some of these individual differences is unclear. While some research works find that men tolerate shift work better than women, others find the opposite [12]. The differences may be explained by men and women being employed in different types of occupations and not by gender.

It is unclear if this individual susceptibility is relevant in other types of work arrangements than night/rotating shifts, such as morning, evening or day shifts. In a study on nurses, it was found that individual differences were related to sleep-related shift work tolerance in day, evening and night shifts, especially night shift [14]. This difference still needs to be compared across other occupations. Shift work tolerance has been most studied in the health sector. However, there are some studies investigating it in other occupational sectors such as oil and gas workers [15] or police officers [16]. However, there are no previous studies comparing shift work tolerance across different occupations.

In the present study, we investigate shift work tolerance among police employees and retail workers. We chose these occupations because we wanted to compare occupations with different requirements, content and population characteristics. On a theoretical level, the two occupations can be grouped into two distinct occupational types attracting individuals with different skills, interests and dispositions [17,18]. Working as a police officer requires a 3-year bachelor’s degree [19]. Enrolment requires successful completion of a selection process. Employees in the retail industry, on the other hand, may have a variety of different educational backgrounds, ranging from high education in leadership to no educational background after high school. Many retail workers have low education or are studying at the same time, and many work only part time [20]. On the basis of this difference, one could assume that workers employed in the retail industry are more diverse than police employees; hence, individual differences are more important for shift work tolerance for retail workers compared to police employees.

The challenges experienced in different occupations that may be relevant for shift work tolerance vary greatly. Fulfilment of basic needs of autonomy, competence and relatedness are universal and important for individuals across contexts and domains [21]. The need for autonomy implies that people have a universal urge to be causal agents and to experience volition [22]. The need for competence concerns people’s inherent desire to be effective in dealing with the environment [23] and the need for relatedness or belongingness reflects the universal propensity to interact with, be connected to, and experience caring for other people [24]. We have previously found that self-determination factors are positively related to good shift work tolerance [25]. Thus, they may be especially important to investigate in relation to shift work tolerance across occupations.

The main aim of this study is to clarify how shift work tolerance, and individual differences in shift work tolerance, differ across occupations, work arrangements and sex. This will make us one step closer to clarifying the nature of shift work tolerance and provide a basis for future longitudinal studies that can investigate tailored interventions to ameliorate different types of circadian disruption.

The study will investigate these specific research questions: how do symptoms of shift work intolerance differ between occupations, sex and work schedule? How do patterns of relations between personality, work factors and shift work intolerance differ between occupations?

Hypothesis: First hypothesis: differences in symptoms of shift work intolerance will be larger between police versus retail workers than between men and women. Second hypothesis: individuals engaged in evening work will have the same intolerance to shift work as night workers, and both groups will show more symptoms of shift work intolerance than day workers. Third hypothesis: individual differences and basic needs will determine more symptoms of shift work intolerance for retail workers than for police employees.

## Methods

The study consists of data from two samples. Sample 1 was recruited from the member pool of The Norwegian Union of Commerce and Office Employees, more specifically workers employed in retail. For Sample 2, all employees in a police district in Norway were invited to participate. The total sample consisted of 727 participants. Sample 1 (retail workers) included 267 women (84%) and 48 men (15%), with age range from 18 to 68 (mean 39 years, standard deviation [SD] = 11.62). Ninety worked only daytime, 197 worked day and evening or only evening, while 21 worked rotating shift work. Sample 2 (police employees) included 199 women (48 %) and 211 men (52%) age ranged from 20 to 68 (mean 41 years, SD = 11.17). In this sample, 169 worked only daytime, 25 worked day and evening, while 195 worked rotating shift work. Daywork is work happening between 7 a.m. and 4 p.m., evening work often occurs sometime between 4 p.m. and 11 p.m. Night work is when the majority of work time falls between 10 p.m. and 6 a.m. [2]. Rotating shift work implies changing between day, evening and night shifts in different types of patterns.

The study was approved by the Norwegian Regional Committee for Medical and Health Research Ethics in the Spring of 2018. All participants were adults above the age of 18 and provided electronic informed consent. The data were collected through online questionnaires. In Sample 1, a weblink with information about the study and the questionnaire was distributed by The Norwegian Union of Commerce and Office Employees to their member through their official Facebook and Twitter accounts in June 2018. Due to the sampling method, no response rate could be attained for this sample. In Sample 2, the questionnaires and information were distributed by e-mail to all employees in a police district in Norway in October 2018. The response rate for this sample was 40% (*N* = 410). Missing values on one or more variables were removed listwise from both samples when performing the analyses.

The participants answered questionnaires including items and instruments that measured demographic and background variables, psychosocial work factors, basic need satisfaction, personality, sleep and health, among others. The work schedule was categorized into either (1) daytime work only, (2) daytime and evening work or only evening work and (3) rotating shift work. Insomnia was assessed using the Norwegian version of the Bergen Insomnia Scale [26]. Symptoms of anxiety and depression were measured with the 14-item Hospital Anxiety and Depression Scale [27]. Sleepiness was measured with four questions from the Basic Nordic Sleep Questionnaire [28]. Exhaustion was measured with eight corresponding items from the Burnout Assessment Tool [29]. We applied the 60-item NEO Five-Factor Inventory to measure the personality traits of the five-factor model [30]. The Basic Need Satisfaction at Work Scale was used to assess three psychological needs at work: autonomy, relatedness and competence [31].

All analyses were performed in IBM SPSS Statistics, version 28. The shift work intolerance variables were slightly skewed. However, this is often not a problem, due to the central limit theorem stating that the sample means of moderately large samples are often well approximated by a normal distribution even if the data are not normally distributed [32]. First, we performed independent-samples *t*-tests comparing all the mean scores of the shift work intolerance symptoms (insomnia, anxiety, depression, sleepiness and exhaustion), between men and women and between retail workers and police employees. Second, we compared the mean scores of the same variables between day workers only, day and evening workers, and rotating shift workers in a one-way between-groups analysis of variance. Third, we performed a correlation analysis between all the study variables. Then we performed five multiple regressions, one for each of the shift work intolerance variables, to examine how sex, age, work experience, shift type and occupational type, were associated with the symptoms.

Lastly, we performed a series of a total of 10 hierarchical regression analyses, 2 for each of the shift work intolerance symptoms for retail workers and police employees. We excluded all day workers from these analyses as we wanted to examine shifts with day and evening work and rotating shift work. This analysis included age, sex and a dichotomous variable categorizing the participants into either daytime and evening time/only evening time workers (value = 1), or rotating shift workers (value = 2), neuroticism, extraversion, autonomy, competence and relatedness.

## Results

We performed independent-samples *t*-tests that compared the mean scores of shift work intolerance symptoms for police versus retail workers shown in Table 1. These showed significant scores on all shift work intolerance symptoms. Regarding the comparisons between men and women, there were significant differences in all shift work intolerance symptoms, except depression.

**Table 1.** Comparing means (SD) of shift work intolerance symptoms police and HK, men and females, day work only, day and evening work, and rotating shift work

SW intolerance symptom	Police (*n* = 343–358)	Retail (*n* = 285–317)	Effect size HK versus police	Men (*n* = 222–231)	Women (*n* = 404–442)	Effect size men versus women	Day (*n* = 229–249)	Day/evening (*n* = 199-218)	Rotating (*n* = 192–199)	Effect size shift type
Anxiety	4.77 (3.22)**	7.62 (4.60)	0.72	4.82 (4.21)**	6.78 (4.27)	0.48	5.65 (3.90)**	7.95 (4.72)	4.67 (2.93)	0.10
Depression	3.32 (2.94)**	5.66 (3.22)	0.72	4.10 (3.60)	4.58 (3.69)	0.13	3.87 (3.44)**	5.90 (4.06)	3.51 (2.88)	0.08
Sleepiness	2.58 (0.51)*	2.72 (1.02)	0.17	2.52 (0.61)**	2.71 (0.85)	0.25	2.51 (0.71)*	2.76 (0.98)	2.65 (0.58)	0.02
Insomnia	11.60 (8.64)**	15.94 (10.81)	0.45	10.80 (8.21)**	15.02 (10.42)	0.44	11.80 (9.06)**	16.79 (11.25)	11.84 (8.39)	0.06
Exhaustion	2.36 (0.60)**	2.96 (0.87)	0.84	2.38 (0.68)**	2.73 (0.80)	0.46	2.54 (0.69)**	2.94 (0.88)	2.60 (0.77)	0.09

*t*-Tests are used to analyse differences between police and retail workers, men and women; ANOVA is used to analyse differences between day, day and evening workers and rotating shift workers, *n* differed for anxiety, depression, sleepiness, insomnia and exhaustion; therefore, range for *n* is reported.

*P < 0.05. **P <. 001.

A one-way between-groups analysis of variance was conducted to examine the impact of shift type on shift work intolerance symptoms (shown in Table 1). There were significant differences for all shift work intolerance symptoms. *Post hoc* comparisons using Bonferroni test indicated that for insomnia, anxiety and fatigue, there were significant differences between all groups. For sleepiness and depression, only the difference between day and day/evening or rotating was significant. The effect sizes were for the comparison between occupations medium and large, for sex, they were medium, and for shift type, they were small.

Table 2 shows correlations between all study variables, illustrating significant correlations of small–medium magnitude between almost all the demographic variables, individual differences and basic needs and the shift work intolerance variables. Table 3 shows the multiple regression of the association between demographic variables, shift and occupational type, and the shift work intolerance symptoms. Overall, the occupational type was the most important variable for most of the shift work intolerance symptoms, with significant betas ranging from −0.12 (insomnia), −0.28 (anxiety), −0.32 (depression) and −0.34 (exhaustion). However, for insomnia, sex (*b* = 0.17) and day versus shift work (*b* = 0.10) also were significant. For anxiety, age had a negative association (*b* = −0.15) and sex and a positive association (*b* = 0.11), the same was the case for exhaustion: age (*b* = 0.18), sex (*b* = 0.12). Regarding sleepiness, working in retail or as a police employee was not significant, but age was negative (*b* = 0.18), and sex (*b* = 0.11) as well as day versus shift work (*b* = 0.08) had a significant positive association. These significant associations indicated that retail workers, evening and rotating shift workers, females and younger workers have higher scores on the shift work tolerance symptoms.

**Table 2.** Correlations between all study variables in the whole sample (*n* = 576–727)

	1.	2.	3.	4.	5.	6.	7.	8.	9.	10.	11.	12.	13.	14.	15.
1. Occupation	–														
2. Sex	−0.37^**^	–													
3. Age	0.09*	−0.02	–												
4. Work experience	0.25**	−0.04	0.40**	–											
5. Day vs shift work	−0.14^**^	−0.05	−0.25^**^	−0.11**	–										
6. Anxiety	−0.34^**^	.22^**^	−0.19^**^	−0.14**	0.09*	–									
7. Depression	−0.32^**^	0.06	−0.12^**^	−0.14**	0.12^**^	0.68^**^	–								
8. Sleepiness	−0.09*	0.12^**^	−0.20^**^	−0.08*	0.12^**^	0.46^**^	0.45^**^	–							
9. Insomnia	−0.22^**^	.20^**^	−0.12^**^	−0.11	0.12^**^	0.58^**^	0.50^**^	0.58^**^	–						
10. Exhaustion	−0.38^**^	0.21^**^	−0.14^**^	−0.13	0.06	0.52^**^	0.48^**^	0.48^**^	0.42^**^	–					
11. Neuroticism	−0.42^**^	0.21^**^	−0.20^**^	−0.14**	0.06	0.58^**^	0.47^**^	0.39^**^	0.40^**^	0.65^**^	–				
12. Extraversion	.33^**^	−0.10^**^	0.12^**^	−0.15**	0.00	−0.29^**^	−0.39^**^	−0.24^**^	−0.20^**^	−0.46^**^	−0.57^**^	–			
13. Autonomy	0.25^**^	−0.12^**^	0.11^**^	0.09*	−0.10*	−0.42^**^	−0.38^**^	−0.32^**^	−0.28^**^	−0.58^**^	−0.53^**^	0.32^**^	–		
14. Competence	0.17^**^	−0.14^**^	0.10*	0.09*	−0.03	−0.31^**^	−0.32^**^	−0.27^**^	−0.21^**^	−0.45^**^	−0.51^**^	0.39^**^	0.65^**^	−	
15. Relatedness	0.22^**^	−0.12^**^	0.06	0.13**	−0.04	−0.32^**^	−0.36^**^	−0.26^**^	−0.25^**^	−0.44^**^	−0.47^**^	0.43^**^	0.56^**^	0.59^**^	–

*P < 0.05; **P < 0.001.

**Table 3.** Multiple regression analysis for demographic, shift and occupational type variables related to shift work intolerance symptoms in the whole sample (*n* = 594–688)

	Anxiety				Depression			
	Beta	SE	96% CI *B*	*P*	Beta	SE	96% CI *B*	*P*
Sex	0.11*	0.35	[0.289,1.660]	0.005	−0.07	0.31	[−1.129,0.095]	0.098
Age	−0.15**	0.03	[−0.084, −0.026]	< 0.001	−0.07	0.01	[−0.050,0.003]	0.077
Work experience	0.00	0.04	[−0.070,0.078]	0.919	−0.03	0.03	[−0.080,0.052]	0.682
Day or shift work	0.01	0.33	[−0.574,0.723]	0.821	0.05	0.30	[−0.186, 971]	0.184
Retail or police	−0.28**	0.35	[−2.984, 1.622]	< 0.001	−0.32**	0.31	[−2.939−1.723]	< 0.001

	Sleepiness				Insomnia			
	Beta	SE	96% CI *B*	P	Beta	SE	96% CI *B*	P
Sex	0.12*	0.07	[0.063,0.323]	0.004	0.17**	0.82	[−1.869, 5.102]	< 0.001
Age	−0.18**	0.00	[−0.018, −0.007]	< 0.001	−0.08	0.04	[−0.136−0.005]	0.068
Work experience	0.01	0.02	[−0.012,0.016]	0.787	−0.03	0.09	[−0.225,0.119]	0.546
Day or shift work	0.08*	0.06	[0.011,0.260]	0.033	0.10*	0.78	[0.397, 3.488}	0.014
Retail or police	−0.01	0.07	[−0.148,0.113]	0.789	−0.12*	0.82	[−4.015, −0.779]	0.004

	Exhaustion							
	Beta	SE	96% CI *B*	P				
Sex	0.10*	0.22	[0.026, 281]	0.018				
Age	−0.11*	0.07	[−0.013, −0.002]	0.011				
Work experience	0.00	0.00	[−0.013,0.014]	0.955				
Day or shift work	−0.01	0.02	[−0.148,0.102]	0.718				
Retail or police	−0.34**	0.06	[−0.677, −0.415]	< 0.001				

Men = 0; Women = 1; day workers = 1, shift workers (evening or rotating) = 2; retail workers = 1, police employees = 2.

*P < 0.05; **P < 0.001.

In Table 4, we show the results for multiple regression analysis performed separately for retail workers and police employees engaged in evening shift or rotating shift work for all the symptoms of shift work intolerance.

**Table 4.** Multiple regression analysis on variables related to anxiety, depression, sleepiness and exhaustion for police employees and retail workers engaged in evening shifts or rotating shifts.

		Retail workers (*n* = 176)					Police employees (*n* = 173)				
Shift work intolerance symptoms	Predictor variables	Beta	SE	96% CI *B*	*P*	*R* ^2^	Beta	SE	96% CI *B*	*P*	*R* ^2^
Anxiety						0.52*					0.07
	Sex	0.01	0.72	[−1.238, 1.603]	0.800		0.11	0.44	[−0.219, 1.535]	0.141	
	Age	−0.06	0.02	[−0.068,0.020]	0.278		0.02	0.02	[−0.038,0.046]	0.844	
	Evening versus rotating	−0.08	0.87	[−2.992,0.437]	0.143		−0.01	0.73	[−1.562, 1.324]	0.871	
	Neuroticism	0.65**	0.42	[2.783, 4.423]	<0.001		0.23*	0.43	[0.217, 1.909]	0.014	
	Extroversion	0.10	0.47	[−0.204, 1.643]	0.126		0.18	0.54	[−0.029, 2.106]	0.057	
	Autonomy	−0.21*	0.29	[−1.352, −0.204]	0.008		−0.07	0.31	[−0.853,0.358]	0.421	
	Competence	0.10	0.34	[−0.290, 1.040]	0.267		0.03	0.32	[−0.526,0.753]	0.726	
	Relatedness	−0.07	0.28	[−0.845,0.277]	0.319		−0.00	0.33	[−0.665,0.647]	0.979	
Depression						0.43**					0.03
	Sex	−0.10	0.67	[−2.444,0.219]	0.101		−0.10	0.45	[−1.463,0.314]	0.204	
	Age	−0.07	0.02	[−0.065,0.018]	0.262		0.09	0.02	[−0.020,0.066]	0.294	
	Evening versu rotating	−0.08	0.81	[−2.761,0.453]	0.158		−0.04	0.74	[−1.826, 1.099]	0.624	
	Neuroticism	0.28**	0.39	[0.563, 2.100]	<0.001		0.10	0.43	[−0.380, 1.334]	0.273	
	Extroversion	−0.16*	0.44	[−1.858, −0.127]	0.025		0.07	0.55	[−0.658, 1.505]	0.441	
	Autonomy	−0.20*	0.27	[−1.164, −0.088]	0.023		0.01	0.31	[−0.578,0.649]	0.908	
	Competence	−0.01	0.32	[−0.657,0.589]	0.914		0.00	0.33	[−0.639,0.658]	0.977	
	Relatedness	−0.15*	0.27	[−1.054, −0.002]	0.049		−0.05	0.34	[−0.846,0.484]	0.591	
Sleepiness						0.35*					0.03
	Sex	0.15*	0.18	[0.052,0.778]	0.025		.01	.08	[−0.139,0.167]	0.856	
	Age	−0.12	.01	[−0.022,0.001]	0.064		−0.06	.00	[−0.011,0.004]	0.418	
	Evening versu rotating	0.05	0.22	[−0.252,0.623]	0.404		−0.02	.13	[−0.288,0.214]	0.772	
	Neuroticism	0.41**	0.11	[0.286,0.705]	<0.001		0.09	0.08	[−0.074,0.221]	0.328	
	Extroversion	0.01	0.12	[−0.209,0.263]	.823		−0.01	0.09	[−0.198,0.172]	0.891	
	Autonomy	−0.31**	0.07	[−0.394, −0.100]	0.001		0.09	0.05	[−0.050,0.164]	.297	
	Competence	0.10	0.09	[−0.081,0.259]	0.302		−0.13	0.06	[−0.195,0.035]	.173	
	Relatedness	−0.00	0.07	[−0.146,0.141]	0.976		−0.00	0.06	[−0.122,0.113]	0.942	
Insomnia						0.32*					0.06
	Sex	0.10	0.07	[−1.161, 7.153]	0.157		0.14	1.24	[−0.034, 4.887]	0.053	
	Age	−0.03	2.54	[−0.165,0.092]	0.573		0.03	.06	[−0.098,0.145]	0.703	
	Evening v rotating	−0.05	1.22	[−6.790, 3.244]	0.486		−0.09	2.05	[−6.541, 1.534]	0.223	
	Neuroticism	0.39**	1.37	[2.856, 7.654]	<0.001		0.17	1.20	[−0.046, 4.696]	0.055	
	Extroversion	0.04	0.85	[−1.989, 3.417]	0.603		0.12	1.51	[−0.843, 5.107]	0.159	
	Autonomy	−0.36**	0.99	[−4.897, −1.537]	<0.001		0.07	0.87	[−0.996, 2.441]	0.408	
	Competence	0.15	0.83	[−0.509, 3.383]	0.147		0.02	0.94	[−1.641, 2.055]	0.825	
	Relatedness	−0.03	2.36	[−1.950, 1.334]	0.712		−0.04	0.96	[−2.312, 1.473]	0.663	
Exhaustion						0.53					0.41**
	Sex	0.14*	0.14	[0.056,0.624]	0.019		0.09	0.07	[−0.028,0.248]	0.118	
	Age	−0.07	0.00	[−0.014,0.004]	0.258		0.00	0.00	[−0.007.007]	0.980	
	Evening versu rotating	0.01	0.17	[−0.292,0.374]	0.807		0.06	0.12	[−0.118,0.336]	0.343	
	Neuroticism	0.33**	0.08	[0.198,0.527]	<0.001		0.42**	0.07	[0.284,0.550]	<0.001	
	Extroversion	−0.13	0.09	[−0.353,0.013]	0.069		−0.13	0.09	[−0.330,0.005]	0.057	
	Autonomy	−0.48**	0.06	[−0.443, −0.217]	<0.001		−0.23**	0.05	[−0.260, −0.067]	0.001	
	Competence	0.03	0.07	[−0.105,0.157]	0.696		−0.07	0.05	[−0.154,0.054]	0.345	
	Relatedness	0.05	0.06	[−0.076,0.148]	0.523		0.04	0.05	[−0.077,0.136]	0.589	

*P < 0.05; **P < 0.001.

For retail workers, the model explained 52% of the variance in anxiety. Neuroticism was positively (*b* = 0.65**) and autonomy negatively (*b* = 0.21*) related to anxiety. Regarding depression, neuroticism was positively (*b* = 0.28**) and extraversion (*b* = 0.16*), autonomy (*b* = 0.20*) and relatedness (−0.52*) negatively related to depression, explaining 43% of the variance. For sleepiness, sex (positively; *b* = 0.15*), neuroticism (positively; *b* = 0.41**) and anutonomy (negatively; *b* = 0.31**) explained 35% of the variance.

The model explained 32% of the variance in insomnia. Neuroticism was positively (*b* = 0.39**) and autonomy negatively (*b* = −0.36**), significantly related to insomnia. The regression model explained 53% of the variance in exhaustion. The contributing variables were sex (positively; *b* = 0.14*), neuroticism (positively; *b* = 0.33**) and autonomy (negatively; *b* = -0.48**).

For police employees, the model explained only 7% of the variance in anxiety, neuroticism was positively (*b* = 0.23*) related to anxiety. None of the variables in the model explained any of the variances in depression, sleepiness or insomnia. The model explained 41% of the variance in exhaustion. Neuroticism was positively (*b* = 0.42**) and autonomy negatively (−0.23*) related to exhaustion.

## Discussion

The findings indicate that the type of occupation is essential for several shift work intolerance variables, in many cases, even more so than gender and shift work arrangement. Furthermore, we found that individual differences are more important in explaining shift work tolerance for retail workers compared to police. This questions the way shift work tolerance is measured as well as the use of demographic and individual differences in designing interventions for shift workers.

The results showed that the differences in symptoms of shift work intolerance were larger between police versus retail workers than between men and women, supporting our first hypothesis. Still, there were also consistent differences in all the shift work intolerance variables except depression between men and women, with women having poorer shift work tolerance than men, in line with previous research [10]. Police workers had better shift work tolerance than retail workers. This may be due to a selection effect, where police workers are selected to be specifically robust. Also, some of the differences between police and retail workers may be due to that there are more women employed as retail workers than police employees. Still, also about half of the police sample were women.

Regarding the shift type, as expected in our second hypothesis, the symptoms of shift work intolerance were higher in evening work and rotating shift work compared to day work. However, we did not expect that the symptoms of shift work intolerance in many cases were higher among evening shift workers compared to night workers. One explanation for this may be that many of the night workers were police workers, who, have better shift work tolerance than retail workers. Also, the content of the work may be more exciting and engaging on the night shift compared to the evening shift. Lastly, evening work may be followed by quick returns, which implies going back to work with less than 11 hours of rest, which in some studies show a more detrimental impact on health than night shifts [33, 34].

The individual differences and basic needs, especially neuroticism and autonomy, had stronger relations to shift work intolerance for retail workers than police employees supporting our third hypothesis. Also, in previous research, neuroticism [35] and autonomy [25] have been the most important factors related to shift work tolerance out of other individual differences and basic needs. However, although we expected personality and basic needs to be more strongly related to shift work tolerance among retail workers compared to police employees, we did not expect that the relations would be not significant for the police employees at all. A recent review also find no associations between the dispositional variables resilience and hardiness and health-related variables among police [36].

This can be because police employees are more similar to the retail workers due to the different educational and selection demands required to take on the jobs. Also, the police employees may have so low scores on neuroticism that it does not make a difference in this context.

The main limitation of the present study is that it is cross-sectional. Hence, we cannot make any predictions of what contributes to better or poorer shift work tolerance. However, as our aims were more descriptive in nature, we have accomplished describing shift work tolerance in different occupations, shift types, and between men and women. The response rate was acceptable, but somewhat low for the police, and uncertain for the retail workers, which may cause response bias issues. Since all the variables were measured with a questionnaire, the findings can be susceptible to common method bias [37]. Also, the non-existence of an established way to measure shift work tolerance is a limitation with previous research as well as the present study. Still, the use of established and validated instruments which are also used in other shift work tolerance research is a strength.

Our study contributes to the field of defining shift work tolerance. Our results indicate that the work schedule (day or shift work) seems to be more associated with insomnia and sleepiness than anxiety, depression and exhaustion. For the latter variables, the type of occupation was more determining. This suggests that the sleep and sleepiness part of the shift work tolerance definition may be assessed well enough with the validated insomnia and sleepiness questionnaires used in the present study. However, we might need to use other measures of the ‘changes in aggression and sensitivity’ part of the definition [7], besides HADS. We need more knowledge on the aggression and sensitivity part of shift work tolerance, and whether this truly is related to sleep or circadian consequences of shift work. Perhaps, potential solutions to test in future research are assessments related to affect regulation, which may measure changes in aggression and sensitivity more accurately than general measures of distress.

Our results serve as a basic guideline for different considerations that should be taken when tailoring work schedules and work demands to individuals in different types of occupations. It is important to show caution using sex differences when adapting shift work to individuals, and also considering the type of work to be done. We have shown that the relationship between individual differences and basic needs on one side and shift work intolerance symptoms on the other side differ in two occupations. These differences also need to be examined across other shift work populations. Some workers, for example, doctors, may experience similar levels of autonomy as the police workers, but be different in other ways. Similarly, nurses have the same sex distribution as the retail workers in our sample. However, they have different educational backgrounds and work contents than retail workers. Future research and practice need to consider the type of occupation, work content, individual characteristics of the workers, and the way shift work tolerance is measured when designing new studies and interventions.
